# Genome assemblies for *Pyricularia* species and related genera isolated from diverse host plants

**DOI:** 10.1128/mra.00091-25

**Published:** 2025-11-24

**Authors:** Avery Meyer, Bram Dutch, Ashab Ahmed, Anna Baloh, Matt Bartholomai, John Boggess, Amelia Burnett, Colin Carver, Evan Courtwright, Chase Eastham, Darrin Egan, Knox Garland, Kelly Claire Gray, Audrey Harper, Megan Johar, Tanner Jones, Leighanne Lyvers, Jacob Marquez, Summer McCune, Matt Mitchell, Carson Moylan, Luke Olsen, Tucker Overton, Oluwatofunmi Oyetan, Khumbo Phiri, Kevin Ramirez, Gavin Robinson, Connor Talbot, Bruno Athie Teruel, Parker Thompson, Lark Wuetcher, Wesley Yang, Andrew Tapia, Jerzy Jaromczyk, Mark Farman

**Affiliations:** 1 Computer Sciences Program, University of Kentucky 4530, Lexington, Kentucky, USA; 2 Department of Computer Sciences, University of Kentucky 4530, Lexington, Kentucky, USA; 3 Department of Horticulture, University of Kentucky 4530, Lexington, Kentucky, USA; 4 Agricultural and Medical Biotechnology Program, University of Kentucky 4530, Lexington, Kentucky, USA; 5 Department of Plant Pathology, University of Kentucky 4530, Lexington, Kentucky, USA; University of California Riverside, Riverside, California, USA

**Keywords:** whole-genome shotgun, blast fungus

## Abstract

We report the availability and preliminary analyses of genomic resources for 35 fungal strains representing *Pyricularia* and related genera. These data will provide new insights into genome expansions, as well as mechanisms driving colonization of new plant hosts.

## ANNOUNCEMENT

The ascomycete fungus *Pyricularia oryzae* causes blast diseases in rice and wheat but also infects diverse Gramineae (1, 2). Recently, we discovered novel host-specialized populations of *P. oryzae* in Brazil (3) and determined that admixture has significantly influenced host range expansion (4). The objective of this study was to develop genomic resources for understudied *Pyricularia* population/species and related genera to fill critical gaps in the species’ evolutionary history.

Fungal strains, whose species affiliations were previously ascertained using sequence-based markers (3, 5–7), were provided by other researchers or isolated from leaf lesions. Cultures were established on oatmeal agar, single spored on 3% water agar, and grown in liquid complete medium with shaking (8). DNA was extracted using a phenol/chloroform method and purified with the Genomic Clean and Concentrator kit (Zymo Inc.) (8). Library construction used a 60-minute tagmentation step but otherwise followed the Illumina DNA Prep protocol (Illumina Inc.). Sequences (150-bp paired-end) were generated using the NovaSeq 6000 platform (Novogene Corp. Inc.).

Undergraduates in an “Applied Bioinformatics” class taught by Farman and Jaromczyk performed the data processing which is detailed in a GitHub repository (9). Sequence quality was assessed with FastQC v0.11.9 (10), and trimming/adaptor removal was performed using Trimmomatic v0.38 (11). VelvetOptimiser v2.2.6 was used to identify optimized assemblies by testing k-mer values between 69 and 129. Genome completeness was evaluated using BUSCO v5.7.0 (odb10_ascomycota database) (12). Annotation utilized MAKER v2.31.11 (13) to perform *de novo* gene predictions with SNAP v2.68.4 (14) and AUGUSTUS v3.4.0 (15) using a custom hidden Markov model. Gene modeling incorporated alignments to reference proteins from *P. oryzae* (9) and the nr database. Putative secreted proteins were identified using SignalP v5.0 (16). Single-nucleotide polymorphisms (SNPs) were called using iSNPcaller (17) based on BLAST alignments between each genome assembly and the B71 genome reference (GCA_004785725.2). Fasta alignment files were generated using custom scripts. Phylogenetic analyses were performed using RAxML 8.2.12 (18) with the GTRCAT substitution model, and bootstrap replications were determined using the autoMRE function. Trees were plotted using ggtree and ggtreeExtra (19, 20).

**Fig 1:**
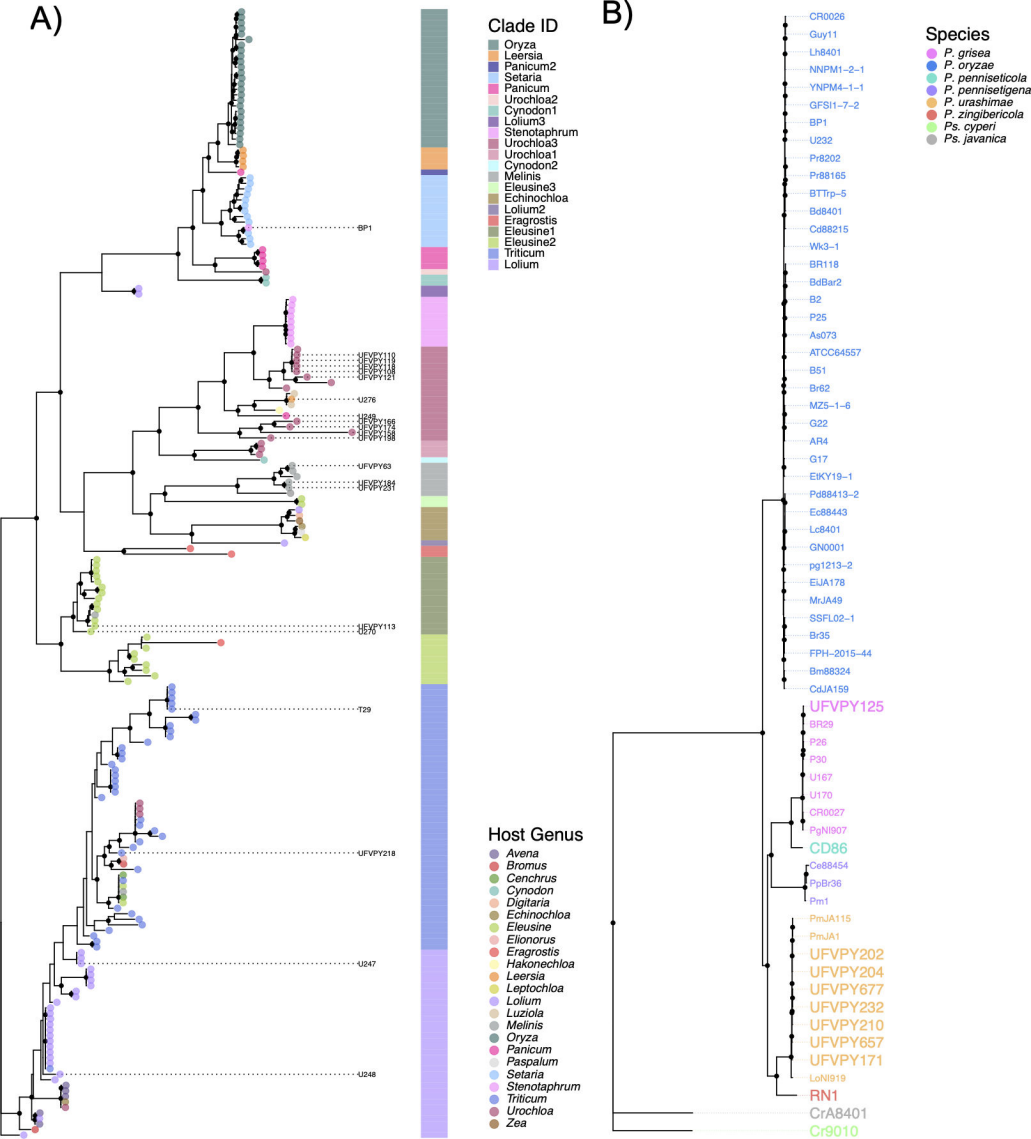
Phylogenetic trees for *Pyricularia oryzae* and other species. (**A**) Tree showing phylogenetic placement of *P. oryzae* isolates sequenced in this study (labeled tips) relative to reference isolates (tip points). Tip/label colors show host-of-origin, and the strip colors distinguish main phylogenetic clades. (**B**) Tree showing *Pyricularia/Pseudopyricularia* species with tip labels colored by species. Larger labels indicate isolates sequenced in this study.

Assembly metrics are summarized in Table 1. Genome sizes ranged from 32. 7Mb for *Pseudopyricularia javanica* to 44.9Mb for *Pyricularia urashimae* isolate UFVPY184, with all BUSCO scores exceeding 96%. GC content varied from 49.1% to 53.6%, and the number of predicted genes ranged from 10,465 (*Pseudopyricularia cyperi*) to 14,617 (*P. oryzae*), with approximately 12.5% being classified as candidate secreted proteins. A phylogenetic tree based on 327,024 SNPs (Fig. 1) indicated that 20 of 23 *P*. *oryzae* isolates clustered according to their respective hosts of origin—consistent with *P. oryzae*’s status as a host-specialized pathogen (3). The three exceptions were U249 and U276—reportedly from *Panicum* and *Leersia,* respectively—which clustered with *Luziola* pathogens inside the Urochloa3 clade, and BP1 from *Stenotaphrum*, which grouped with the *Setaria* pathogens. A tree built using 2,223,388 SNPs in the non-*P*. *oryzae* isolates (Fig. 1) also showed grouping patterns consistent with host-of-origin. UFVPY125 from *Digitaria* grouped with *Pyricularia grisea*, and the isolates from *Urochloa* and *Panicum maximum* belonged to *P. urashimae*. Due to the absence of reference genomes, the others occupied singleton branches whose lengths revealed high divergence from *P. oryzae* (≥ 10%), supporting these strains’ prior classifications as distinct *Pyricularia* or *Pseudopyricularia* species (*Pyricularia penniseticola*, *Pyricularia zingibericola*, *Ps. javanica,* and *Ps. cyperi*) (6, 21).

**TABLE 1:** Genome assembly statistics

Sample name	Species	Host	Genome accession	SRA accession	Genome size (bp)	# Contigs	N50 (bp)	Coverage	%GC	BUSCO score	# Predicted proteins	# Secreted proteins	Reference
BP1	*Pyricularia oryzae*	*Stenotaphrum secundatum*	JASWIG000000000	SRR23400923	44,269,911	8,402	38,801	28	50.7	96.6%	14,617	1,775	(3)
CD0086	*Pyricularia penniseticola*	*Pennisetum typhoides*	JAVHJQ000000000	SRR23499064	37,718,974	9,300	9,584	41	50.8	97.8%	11,816	1,425	(6)
Cr9010	*Pseudopyricularia cyperi*	*Cyperus rotundifolia*	JASAOQ000000000	SRR23498685	35,206,045	7,256	18,270	28	52.1	97.3%	10,465	1,223	(7)
CrA8401	*Pseudopyricularia javanica*	*Cyperus rotundifolia*	JARHUK000000000	SRR23199414	32,715,629	5,506	17,480	53	53.6	98.0%	10,631	1,244	(7)
RN001	*Pyricularia zingibericola*	*Zingiber officinalis*	JASWIJ000000000	SRR23476220	37,907,990	6,067	24,913	57	49.9	96.2%	10,856	1,306	(6)
T21	*Pyricularia oryzae*	*Triticum aestivum*	JAVHTW000000000	SRR23388169	43,329,010	2,709	88,629	24	49.9	98.0%	12,571	1,551	(3)
T29	*Pyricularia oryzae*	*Triticum aestivum*	JBMKKG000000000	SRR27892070	42,317,924	1,784	64,165	37	50.0	98.1%	12,983	1,686	(3)
T6	*Pyricularia oryzae*	*Triticum aestivum*	JASWIK000000000	SRR23501853	42,058,027	2,590	55,184	102	50.1	96.9%	13,375	1,781	(3)
U247	*Pyricularia oryzae*	*Festuca arundinacea*	JBCEWT000000000	SRR27892087	44,649,226	6,941	66,992	87	50.0	98.0%	13,610	1,788	(3)
U248	*Pyricularia oryzae*	*Lolium multiflorum*	JBIYZY000000000	SRR28533131	43,867,247	4,388	67,659	84	50.0	97.6%	13,723	1,717	(3)
U249	*Pyricularia oryzae*	*Panicum sabulorum*	JBCARD000000000	SRR28533132	40,914,617	3,426	104,435	88	50.8	98.4%	13,049	1,751	(3)
U270	*Pyricularia oryzae*	*Eleusine indica*	JAVHUH000000000	SRR23567522	43,877,699	7,125	13,674	35	49.9	96.9%	14,580	1,793	(3)
U276	*Pyricularia oryzae*	*Leersia hexandra*	JAUKTU000000000	SRR23388404	41,408,883	7,474	18,175	41	50.7	98.2%	13,832	1,759	(3)
UFVPY108	*Pyricularia oryzae*	*Urochloa brizantha*	JAVHTU000000000	SRR23491016	39,330,571	6,352	17,944	21	51.7	98.1%	13,521	1,770	(3)
UFVPY110	*Pyricularia oryzae*	*Urochloa brizantha*	JAVHTV000000000	SRR23498602	40,155,654	3,820	38,087	30	51.6	98.1%	13,487	1,764	(3)
UFVPY113	*Pyricularia oryzae*	*Eleusine indica*	JBEURV000000000	SRR27893664	43,332,199	1,906	70,336	58	49.9	98.0%	13,243	1,753	(3)
UFVPY118	*Pyricularia oryzae*	*Urochloa brizantha*	JBPCDH000000000	SRR23385021	40,034,794	4,807	25,618	71	51.7	98.4%	13,551	1,759	(3)
UFVPY119	*Pyricularia oryzae*	*Urochloa brizantha*	JAVHSR000000000	SRR23386668	40,329,958	3,686	44,150	88	51.5	98.5%	13,488	1,783	(3)
UFVPY121	*Pyricularia oryzae*	*Urochloa brizantha*	JASWIF000000000	SRR23387524	40,535,769	3,922	38,129	58	51.5	97.9%	13,580	1,784	(3)
UFVPY125	*Pyricularia grisea*	*Urochloa brizantha*	JASWII000000000	SRR23456574	41,712,136	5,154	55,303	71	49.4	96.9%	11,942	1,551	(3)
UFVPY158	*Pyricularia oryzae*	*Urochloa brizantha*	JAVHTT000000000	SRR23455553	36,015,173	11,335	5,656	36	52.0	97.9%	14,397	1,503	(3)
UFVPY166	*Pyricularia oryzae*	*Urochloa brizantha*	JBJXVL000000000	SRR27893665	39,680,865	3,154	84,342	82	50.7	97.7%	12,750	1,722	(3)
UFVPY171	*Pyricularia urashimae*	*Urochloa brizantha*	JASWIH000000000	SRR23388381	39,770,362	1,978	118,202	41	51.0	97.3%	12,320	1,766	(3)
UFVPY174	*Pyricularia oryzae*	*Urochloa brizantha*	JAVHTX000000000	SRR23404000	37,458,192	1,835	57,082	52	51.8	98.0%	11,897	1,477	(3)
UFVPY184	*Pyricularia oryzae*	*Melinis repens*	JBCNYJ000000000	SRR27893520	44,926,346	5,626	62,152	90	49.2	98.0%	13,231	1,749	(3)
UFVPY198	*Pyricularia oryzae*	*Urochloa brizantha*	JBMNVX000000000	SRR27918925	38,944,860	1,755	98,072	82	51.0	98.2%	12,566	1,744	(3)
UFVPY202	*Pyricularia urashimae*	*Urochloa brizantha*	JBFYDN000000000	SRR27891905	42,200,512	2,052	76,417	69	50.4	98.0%	12,729	1,770	(3)
UFVPY204	*Pyricularia urashimae*	*Urochloa brizantha*	JBCAWL000000000	SRR27918216	41,269,248	1,859	77,297	56	50.4	98.0%	12,429	1,763	(3)
UFVPY210	*Pyricularia urashimae*	*Panicum maximum*	JBCARE000000000	SRR28051826	41,100,774	1,892	80,230	64	50.5	98.0%	12,338	1,747	(3)
UFVPY218	*Pyricularia oryzae*	*Triticum aestivum*	JBCEYJ000000000	SRR27891917	43,094,738	5,694	34,577	48	50.5	97.7%	13,682	1,782	(3)
UFVPY231	*Pyricularia oryzae*	*Melinis repens*	JBCASK000000000	SRR27893663	44,428,863	2,078	101,661	67	49.2	98.3%	12,925	1,762	(3)
UFVPY232	*Pyricularia urashimae*	*Urochloa brizantha*	JBCEVS000000000	SRR27891887	41,802,667	1,976	83,582	63	50.5	98.0%	12,223	1,693	(3)
UFVPY63	*Pyricularia oryzae*	*Melinis repens*	JBBULU000000000	SRR27892088	43,402,530	2,270	75,381	79	49.1	97.7%	12,646	1,676	(3)
UFVPY657	*Pyricularia urashimae*	*Urochloa brizantha*	JBDQYV000000000	SRR29065219	43,027,175	3,161	66,750	80	50.3	97.9%	12,455	1,696	(3)
UFVPY677	*Pyricularia urashimae*	*Urochloa brizantha*	JBLIOR000000000	SRR27917628	41,859,759	2,173	87,678	83	50.5	98.3%	12,143	1,692	(3)

These 35 genomes provide valuable resources for studying *Pyricularia* evolution and will offer insights into genetic divergence, host adaptation, and genome dynamics.

## Data Availability

Raw read data and assembled genomes are deposited in NCBI under BioProject PRJNA926786 with the individual accessions listed in Table 1. Genome annotations (GFF files) and variant call data (VCF files) are accessible in Zenodo (22). Detailed methods and custom scripts are provided on GitHub (9).
